# Siglec‐1 inhibits RSV‐induced interferon gamma production by adult T cells in contrast to newborn T cells

**DOI:** 10.1002/eji.201747161

**Published:** 2018-01-18

**Authors:** Jop Jans, Wendy W.J. Unger, Marloes Vissers, Inge M.L. Ahout, Inge Schreurs, Arthur Wickenhagen, Ronald de Groot, Marien I. de Jonge, Gerben Ferwerda

**Affiliations:** ^1^ Laboratory of Pediatric Infectious Diseases Department of Pediatrics Radboud Institute for Molecular Life Sciences Radboud university medical center Nijmegen The Netherlands; ^2^ Laboratory of Pediatrics Erasmus MC‐Sophia Children's Hospital Rotterdam The Netherlands; ^3^ Department of Immune Mechanisms National Institute for Public Health and the Environment Bilthoven The Netherlands

**Keywords:** CD43, Interferon gamma, Newborns, Respiratory syncytial virus, Siglec‐1

## Abstract

Interferon gamma (IFN‐γ) plays an important role in the antiviral immune response during respiratory syncytial virus (RSV) infections. Monocytes and T cells are recruited to the site of RSV infection, but it is unclear whether cell‐cell interactions between monocytes and T cells regulate IFN‐γ production. In this study, micro‐array data identified the upregulation of sialic acid‐binding immunoglobulin‐type lectin 1 (Siglec‐1) in human RSV‐infected infants. In vitro, RSV increased expression of Siglec‐1 on healthy newborn and adult monocytes. RSV‐induced Siglec‐1 on monocytes inhibited IFN‐γ production by adult CD4^+^ T cells. In contrast, IFN‐γ production by RSV in newborns was not affected by Siglec‐1. The ligand for Siglec‐1, CD43, is highly expressed on adult CD4^+^ T cells compared to newborns. Our data show that Siglec‐1 reduces IFN‐γ release by adult T cells possibly by binding to the highly expressed CD43. The Siglec‐1‐dependent inhibition of IFN‐γ in adults and the low expression of CD43 on newborn T cells provides a better understanding of the immune response against RSV in early life and adulthood.

## Introduction

Respiratory syncytial virus (RSV) is a major cause of respiratory disease worldwide. Nearly all infants have been infected by RSV by the age of 2 years. Infants and adults remain susceptible every year to RSV infections with genetically related viruses or identical virus strains 1. The high rate of reinfections by RSV results in a significant burden on health care in infants and elderly 2. Interferon gamma (IFN‐γ) and T cells are essential to mount an adequate immune response during RSV infections in newborn and adult mice 3, 4, 5. This suggests an important role for IFN‐γ in the clearance of primary infections as well as in reinfections 4, 6. Inhibition or inadequate activation of T cells resulting in lower secretion of IFN‐γ may explain this high rate of reinfections 7, 8. Monocytes and T cells are both recruited to the site of RSV infection and the role of monocytes in the pathogenesis of RSV infections has been appreciated for decades 9, 10, 11, 12, 13, 14, 15. RSV increases the expression of major histocompatibility complex (MHC) molecules on monocytes, activates CD4^+^ T cells and induces the production of IFN‐γ by human immune cells In vitro 15. It is unknown whether monocytes exert a regulatory role on effector T cell function via cell‐cell interaction and thus regulate the production of IFN‐γ. In this study, we used an unbiased approach to select membrane‐bound receptors on monocytes that are induced during RSV infection in infants and could play a role in cell‐cell interactions with T cells. We selected acid‐binding immunoglobulin‐type lectin 1 (Siglec‐1) and monitored the induction of Siglec‐1 on monocytes after exposure of newborn and adult immune cells to RSV In vitro. In addition, we determined the role of Siglec‐1 during RSV‐induced release of IFN‐γ by T cells in newborns and adults and investigated the expression of CD43, as the natural ligand for Siglec‐1, on newborn and adult T cells.

## Results

### Induction of Siglec‐1 expression during RSV infection

**Figure 1:**
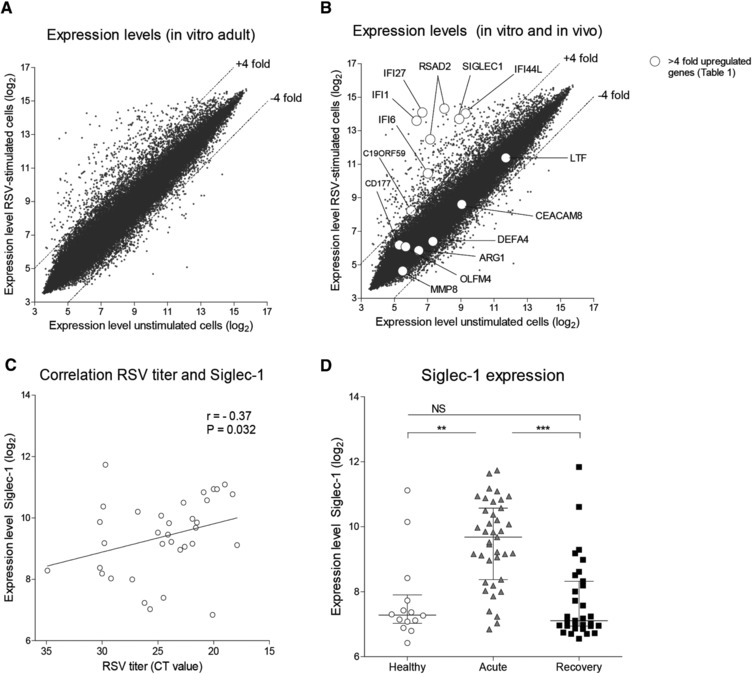
Induction of Siglec‐1 expression during RSV infection. (A) Transcriptome analysis of unstimulated adult PBMCs (*N* = 5) (x‐axis) compared to RSV‐stimulated adult PBMCs (*N* = 5) stimulated with RSV for 24 h (y‐axis). Plots in the upper left corner represent genes that are upregulated during In vitro RSV infection of adult PBMCs. A difference of ± 4‐fold difference between expression levels of unstimulated compared to expression levels of RSV‐stimulated PBMCs was used to determine up‐ and downregulation of genes. Data shown are pooled from two or more independent experiments with two or three samples per experiment (B) Transcriptome analysis of unstimulated adult PBMCs (x‐axis) compared to RSV‐stimulated adult PBMCs (y‐axis) combined with upregulated genes in whole blood from RSV‐infected infants in Table 1 (white dots). (C) Correlation of Siglec‐1 gene expression (y‐axis) and RSV titers in the nasopharyngeal aspirate (x‐axis) during infant RSV infection. (D) Comparison of Siglec‐1 gene expression level in healthy infants (Healthy; *N* = 14), RSV‐infected infants (Acute; *N* = 40) and infants 4–6 weeks after RSV infection (Recovery; *N* = 30). Data points depicted represent individual samples from two or more independent experiments with ten to fifteen samples per experiment Data are represented as median ± interquartile range. Statistical analysis employed Kruskal‐Wallis test and if significant followed by Mann–Whitney U test. Spearman correlation test was used for correlation tests. Dashed lines indicate a 4‐fold difference of expression level between x‐axis and y‐axis. **p<0.01. ***p<0.001.

We first compared whole blood gene expression levels from RSV‐infected infants with healthy infants to select genes that are upregulated during RSV infection in vivo. Fifteen genes were upregulated during in vivo RSV infection using a cut‐off value of 4‐fold difference (Table 1). We next compared In vitro gene expression levels of unstimulated adult PBMCs versus RSV‐stimulated adult PBMCs. More than four hundred genes were upregulated in adult PBMCs during In vitro RSV infection using a cut‐off value of 4‐fold difference (Fig. 1A). Seven genes were upregulated in both the analysis derived from RSV‐infected infants and the analysis derived from RSV‐stimulated PBMCs: *C19ORF59, IFI27, IFI44L, IFI1, IFI6, OASL, RSAD2* and *SIGLEC1* (Table 1; Fig. 1B). Via this approach, we combined transcriptome analyses from RSV‐infected infants with In vitro RSV‐stimulated adult PBMCs and identified potential genes that could be involved in the cell–cell interaction between monocytes and T cells. *SIGLEC1* was the only gene that has been implicated in direct cell‐cell contact with T cells 16 and was therefore selected as our gene of interest for further investigation. We observed a correlation between the RSV titer in the nasopharyngeal aspirate of RSV‐infected infants and expression of Siglec‐1 (Fig. 1C). The expression of Siglec‐1 was higher in infants during the acute phase of infection compared to infants in the recovery phase, which suggests a temporal induction of Siglec‐1 during RSV infection (Fig. 1D). Eight children provided a blood sample during the acute phase and the recovery phase. From these eight individuals, paired analysis showed a reduction of Siglec‐1 expression in all cases (data not shown).

**Table 1:** Upregulation of gene expression in whole blood from RSV‐infected infants. Median gene expression levels in whole blood from RSV‐infected infants (*N* = 40) (row 1) and healthy controls (*N* = 14) (row 2) are depicted. Difference in gene expression was calculated as followed: (median gene expression level RSV‐infected infants) – (median gene expression level healthy controls) (row 3). Differences in gene expression were log_2_‐transformed and genes with ≥ 4‐fold difference between RSV‐infected infants and healthy controls are depicted (row 4)

Gene	Median expression level RSV‐infected infants (*N* = 40)	Median expression level healthy controls (*N* = 14)	Difference expression level (RSV versus healthy)	Log_2_ transformed difference in expression level
**IFI27**	12.03	6.71	5.3	39.4
**CD177**	10.90	7.39	3.5	11.3
**LTF**	10.75	7.98	2.8	7.0
**OLFM4**	8.87	6.03	2.8	7.0
**MMP8**	8.81	6.22	2.6	6.1
**RSAD2**	11.69	9.23	2.5	5.7
**SIGLEC1**	9.68	7.28	2.4	5.3
**IFI44L**	11.56	9.20	2.4	5.3
**ARG1**	9.64	7.38	2.3	4.9
**CEACAM8**	9.01	6.70	2.3	4.9
**C19ORF59**	11.51	9.32	2.2	4.6
**RSAD2**	10.93	8.73	2.2	4.6
**DEFA4**	9.55	7.48	2.1	4.3
**IFI6**	11.86	9.82	2.0	4.0
**IFIT1**	11.94	9.91	2.0	4.0

### RSV induces Siglec‐1 on monocytes

**Figure 2:**
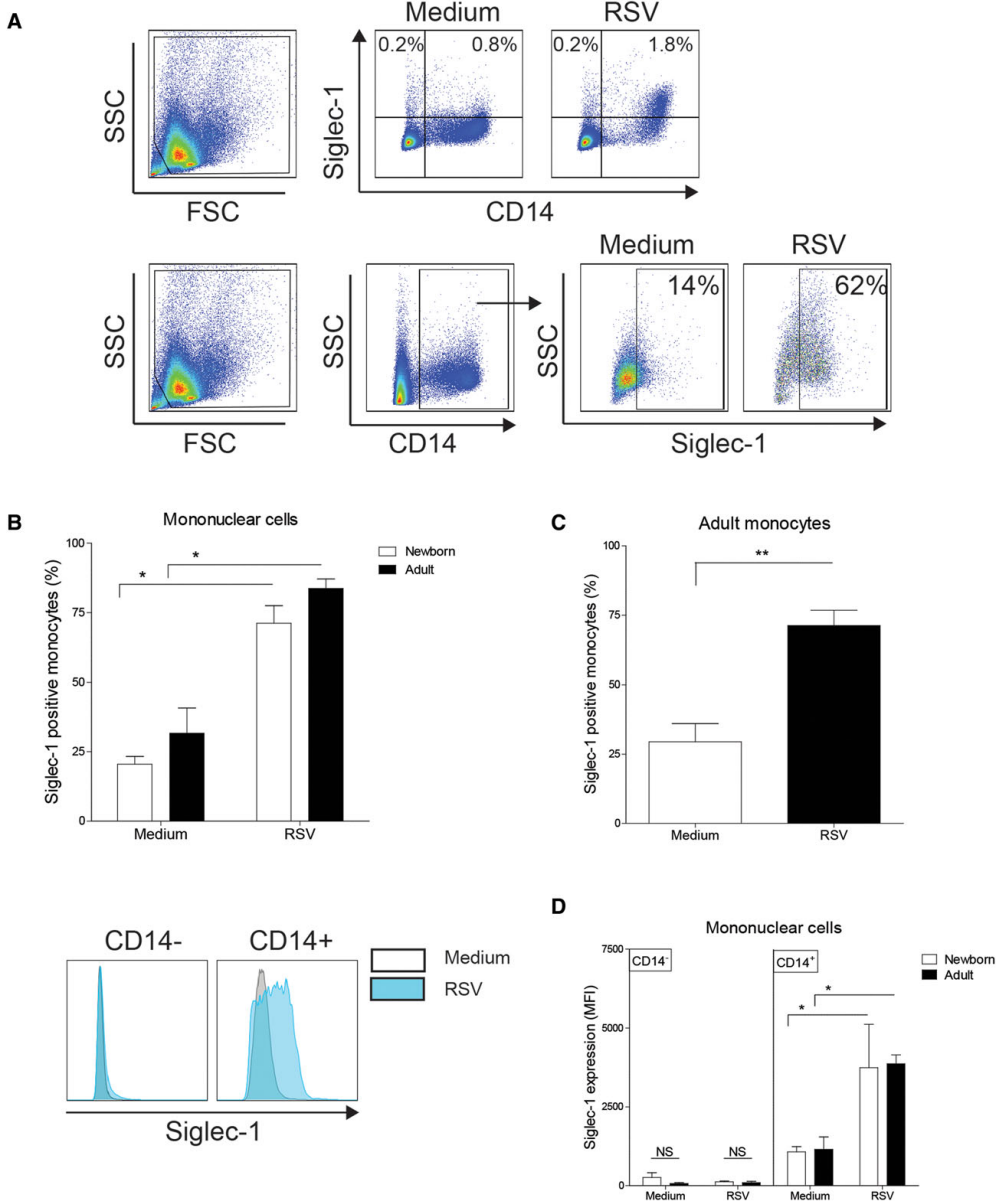
RSV induces Siglec‐1 on monocytes. (A): Representative gating strategy to determine Siglec‐1 expression on monocytes (CD14^+^ cells) after stimulation of MCs with RSV. (B‐C): Percentage of Siglec‐1^+^ monocytes after stimulation of (B) MCs or (C) adult monocytes with RSV N = 5‐7. (D): Mean fluorescence intensity of Siglec‐1 on CD14^−^ and CD14^+^ cells after stimulation of MCs with RSV (*N* = 5). Data represent means ± SEM. Statistical analyses employed the Wilcoxon matched‐pairs signed rank test. *p<0.05. **p<0.01. Data are pooled from two or more experiments with two to four samples per experiment.

We employed newborn CBMCs and adult PBMCs to investigate the induction of Siglec‐1 by RSV In vitro. We observed an increase of Siglec‐1^+^ cells only in the CD14^+^ fraction that consists of monocytes (Fig. 2A, representative figure). The RSV‐induced increase of Siglec‐1^+^ monocytes was observed in newborn and adult (Fig. 2B). Only a small percentage of newborn and adult CD14^−^ cells expressed Siglec‐1 which did not increase after exposure to RSV (Fig. 2A, representative figure). The increase in frequency of Siglec‐1 positive monocytes when stimulating purified adult monocytes with RSV suggests a direct effect of RSV on monocytes (Fig. 2C). In addition, we showed that RSV increased the quantitative expression of Siglec‐1 on newborn and adult CD14^+^ monocytes determined by mean fluorescence intensity (Fig. 2D).

### RSV‐induced IFN‐γ is delayed in newborns and dependent on CD4^+^ T cells

**Figure 3:**
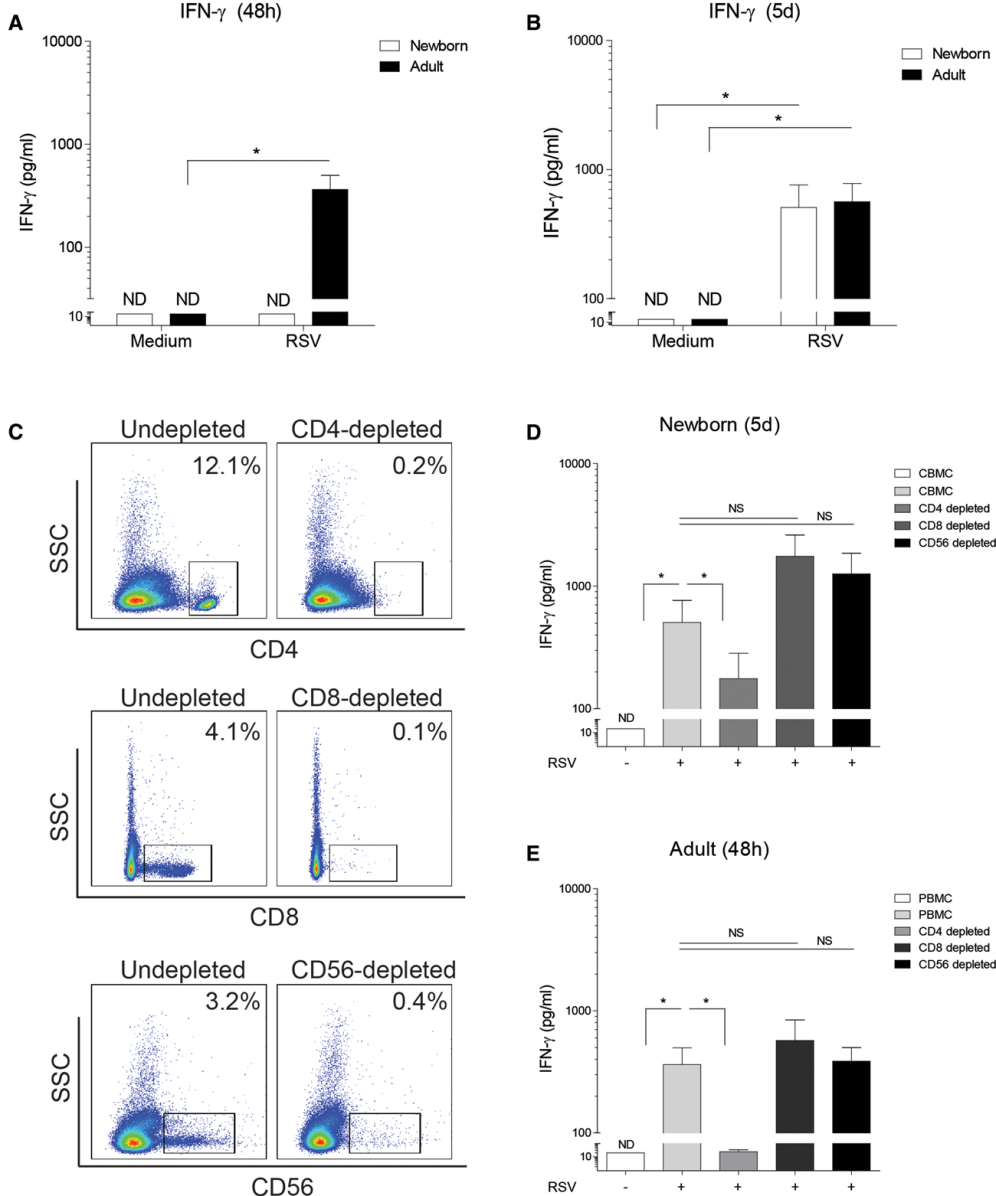
RSV‐induced IFN‐γ is delayed in newborns and dependent on CD4^+^ T cells. (A‐B): Production of IFN‐γ (measured by ELISA performed in duplo) by CBMCs and PBMCs after stimulation with RSV for (A) 48 h or (B) 5 days. *N* = 5. Data are pooled from two or more experiments with two to four samples per experiment. (C): Representative flow cytometry results of MCs after depletion of CD4^+^, CD8^+^ or CD56^+^ cells. (D‐E: RSV‐induced IFN‐γ release, measured by ELISA performed in duplo, by (D) CBMCs, CD4‐depleted CBMCs, CD8‐depleted CBMCs or CD56‐depleted CBMCs after 5 days and (E) RSV‐induced IFN‐γ release by PBMCs, CD4‐depleted PBMCs, CD8‐depleted PBMCs or CD56‐depleted PBMCs after 48 h. *N* = 5. Data are pooled from two or more experiments with two to four samples per experiment. Data represent means ± SEM. Statistical analysis employed Wilcoxon matched‐pairs signed rank test for two conditions and repeated measures ANOVA with Bonferroni's Multiple Comparison Test for more than two conditions. Comparative testing between newborns and adults was performed with Mann–Whitney U test. *p<0.05.

During RSV infection, IFN‐γ is an important cytokine that can be produced by T cells. Newborn CBMCs are naïve and devoid of RSV‐specific memory T cells whereas adults contain RSV‐specific memory T cells 17. Significant amounts of IFN‐γ were measured in the supernatant of adult MCs two days after exposure to RSV, whereas IFN‐γ was not detectable in supernatants from newborn MCs (Fig. 3A). However, RSV induced IFN‐γ release by newborn MCs after an incubation period of 5 days was comparable to the induction of IFN‐γ in adults after 5 days (Fig. 3B). Mononuclear cells were depleted from CD4^+^, CD8^+^ and CD56^+^ cells to discern which cells within the MC fraction produced IFN‐γ in response to RSV (Fig. 3C). Depletion of CD4^+^ cells reduced the RSV‐mediated production of IFN‐γ in cultures of both newborn and adult MCs (Fig. 3D). No reduction of RSV‐induced IFN‐γ release was observed after depletion of CD8^+^ cells and CD56^+^ cells (Fig. 3D).

### RSV‐induced Siglec‐1 inhibits IFN‐γ release by adult T cells but not by T cells from newborns

**Figure 4:**
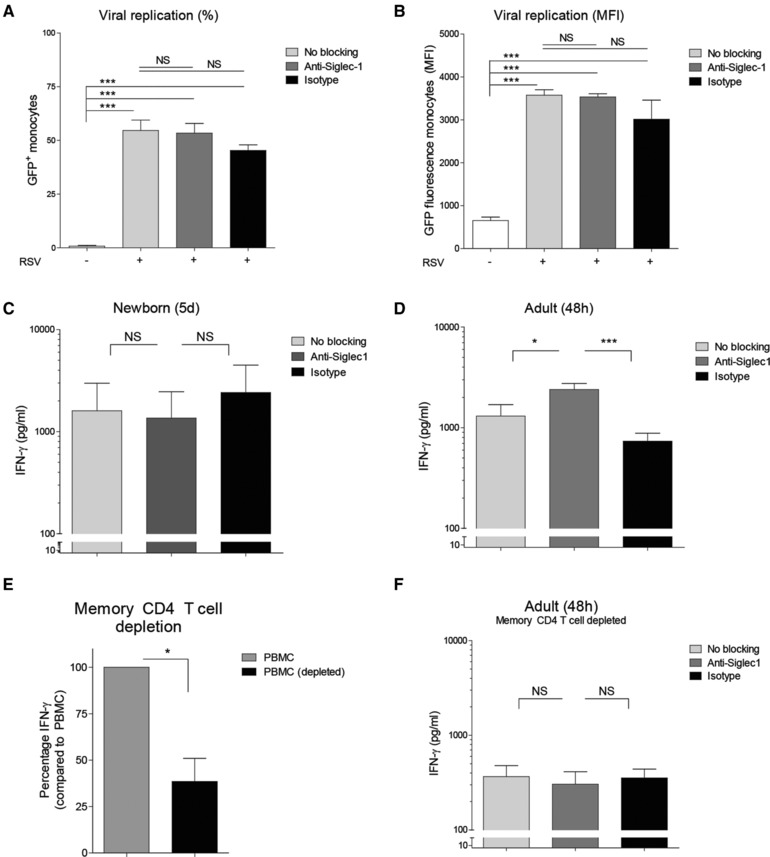
RSV‐induced Siglec‐1 inhibits IFN‐γ release by adult T cells but not by T cells from newborns. A–B: (A) Percentage of RSV^+^ monocytes and (B) GFP fluorescence (MFI) of monocytes after stimulation of adult MCs with RSV in the absence (No blocking) or presence of monoclonal antibodies against Siglec‐1 (Anti‐Siglec‐1) or in the presence of matching isotype controls (Isotype). Data were obtained by flow cytometry. *N* = 5. Data are pooled from two or more experiments with two to four samples per experiment. (C and D): RSV‐induced IFN‐γ release by (C) newborn CBMCs after 5 days or (D) adult PBMCs after 48h in the absence (No blocking) or presence of monoclonal antibodies against Siglec‐1 (anti‐Siglec‐1) or in the presence of matching isotype controls (Isotype). *N* = 4–6. Data are pooled from two or more experiments with two to four samples per experiment performed in duplo. (E:) Percentage of RSV‐induced IFN‐γ by PBMCs or PBMCs depleted from memory CD4^+^ T cells. RSV‐induced IFN‐γ by PBMCS was set to 100. N = 5. Data are pooled from two or more experiments experiments with two to four samples per experiment performed in duplo. (F): RSV‐induced IFN‐γ release by adult PBMCs depleted from memory CD4^+^ T cells after 48h in the absence (No blocking) or presence of monoclonal antibodies against Siglec‐1 (anti‐Siglec‐1) or in the presence of matching isotype controls (Isotype). *N* = 5. Data are pooled from two or more experiments experiments with two to four samples per experiment performed in duplo. Data represent means ± SEM. Statistical analyses employed the Wilcoxon matched‐pairs signed rank test for paired analysis between two conditions and repeated measures ANOVA with Bonferroni's Multiple Comparison Test for paired analysis between more than two conditions. *p<0.05. ***p<0.001.

Siglec‐1 is the only siglec without a signaling motifs which suggests a mode of action via cell‐cell interaction 18. To investigate cell‐cell interactions between monocytes and T cells, we used monoclonal blocking antibodies against Siglec‐1 to determine the effect of Siglec‐1 on the release of IFN‐γ by CD4^+^ T cells. Exposure of MCs to the blocking antibodies alone did not induce IFN‐γ in newborns or adults (data not shown). Blocking Siglec‐1 did not affect the percentage of RSV‐infected adult monocytes or the amount of replication in adult monocytes as depicted by mean fluorescence intensity (Fig. 4A–B). Blocking of Siglec‐1 did not affect RSV‐induced IFN‐γ release by T cells from newborn MCs (Fig. 4C), but, in contrast to newborns, blocking Siglec‐1 significantly increases the RSV‐induced IFN‐γ release by adult MCs (Fig. 4D). To determine whether the inhibitory effect of Siglec‐1 in adult PBMCs was due to the presence of memory CD4^+^ T cells, adult PBMCs were depleted from memory CD4^+^ T cells. Depletion of memory CD4^+^ T cells reduced the production of RSV‐induced IFN‐γ by adults (Fig. 4E), Blocking Siglec‐1 no longer affected RSV‐induced IFN‐γ release by adult T cells when PBMCs are depleted of memory CD4^+^ T cells (Fig. 4F).

### Low CD43 expression on newborn and adult naïve CD4^+^ T cells

**Figure 5:**
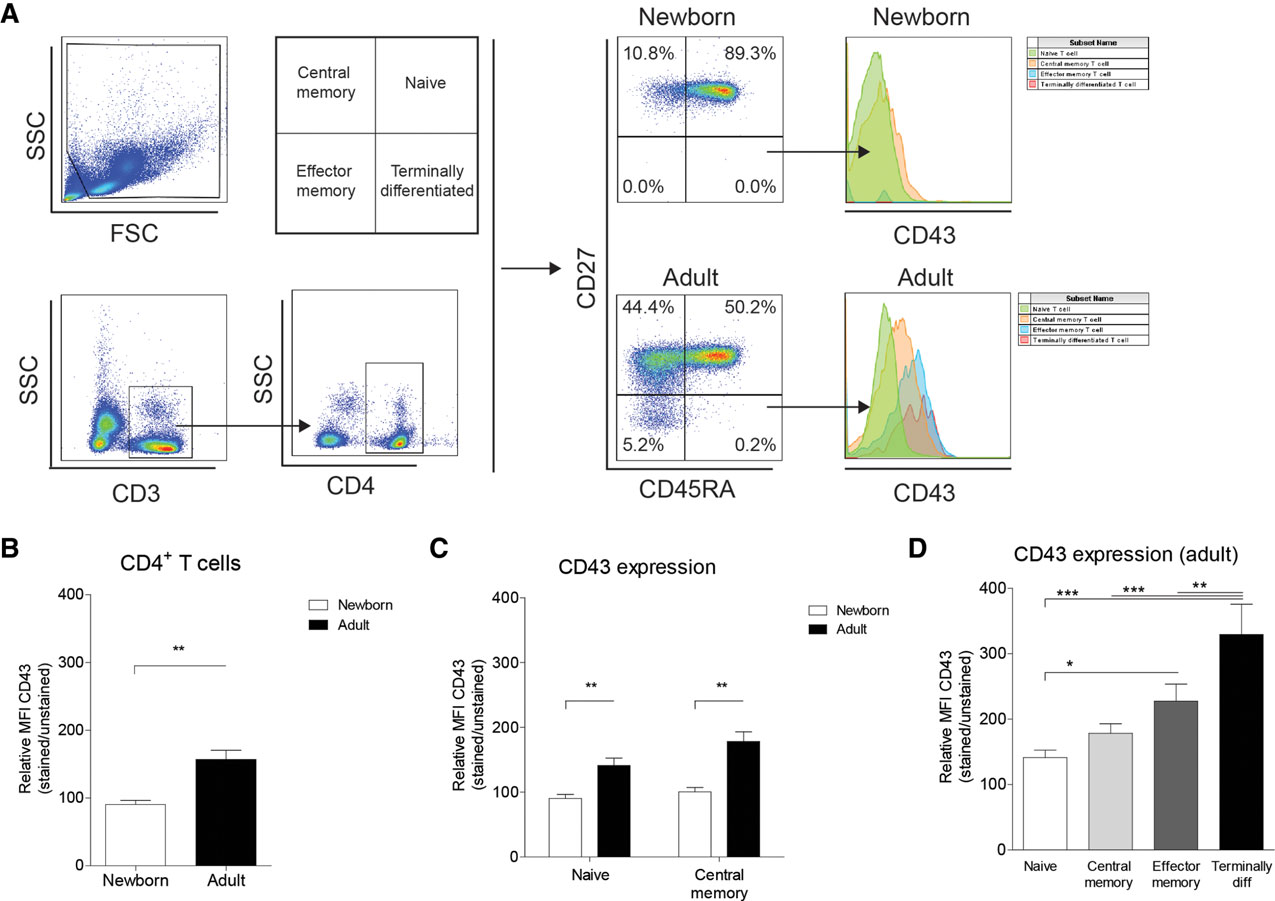
Low CD43 expression on newborn CD4^+^ T cells compared to adults. (A) Representative gating strategy to measure CD43 expression on naïve (CD3^+^CD4^+^CD45RA^+^CD27^+^), central memory (CD3^+^CD4^+^CD45RA^−^CD27^+^), effector memory (CD3^+^CD4^+^CD45RA^−^CD27^−^) and terminally differentiated (CD3^+^CD4^+^CD45RA^+^CD27^−^) CD4^+^ T cells. (B) Expression of CD43 on newborn and adult CD4^+^ T cells. (C) CD43 expression on naïve and central memory CD4^+^ T cells in newborns and adults. *N* = 5–6. D: Expression of CD43 on adult naïve, central memory, effector memory and terminally differentiated CD4^+^ T cells. Data are pooled from two or more experiments with two to four samples per experiment. Data represent means ± SEM. Statistical analysis employed Repeated measures ANOVA with Bonferroni's Multiple Comparison Test for paired analysis between more than two conditions. Comparative testing between newborns and adults was performed with Mann–Whitney U test. *p: <0.05. **p<0.01; ***p<0.001.

Our data showed that despite comparable induction of Siglec‐1 expression in newborn and adult monocytes, Siglec‐1 inhibited IFN‐γ by adult T cells in the presence of memory CD4+ T cells. Siglec‐1 is a sialic‐acid binding receptor and CD43 is a highly sialylated receptor on T cells that acts as a natural ligand for Siglec‐1. We hypothesized that CD43 may be differentially expressed on newborn,, adult naïve and adult memory T cells that would explain the inhibitory effect of Siglec‐1 only when adult memory CD4^+^ T cells are present. The expression of CD43 was higher on adult CD4^+^ T cells compared to newborns (Fig. 5A–B). Additionally, we investigated the expression of CD43 on the major subsets of CD4^+^ T cells, being naïve, central memory, effector memory and terminally differentiated CD4^+^ T cells (Fig. 5A). Adult CD4^+^ T cells consisted of all the measured subsets of CD4^+^ T cells, whereas, as expected, newborn effector memory (average of 0.019%) and terminally differentiated CD4^+^ T cells (average of 0.006%) were barely detected (Fig. 5A). CD43 expression on newborn naïve and newborn central memory CD4^+^ T cells was significantly lower compared to adults (Fig. 5C). Adult effector memory and terminally differentiated CD4^+^ T cells contained a higher expression of CD43 compared to adult naïve CD4^+^ T cells (Fig. 5D).

## Discussion

We demonstrate for the first time that (a) Siglec‐1 is upregulated after RSV infections in vivo and In vitro on newborn and adult monocytes, (b) the RSV‐induced production of IFN‐γ is dependent on CD4^+^ T cells in both adults and newborns but delayed in newborns, and (c) Siglec‐1 inhibits the RSV‐induced IFN‐γ release by adult T cells whereas the production of IFN‐γ by newborn T cells is not affected by Siglec‐1. Finally, we show that (d) the expression of CD43, the natural ligand for Siglec‐1, is lower on newborn CD4^+^ T cells compared to adult CD4^+^ T cells.

In this study, a micro‐array data base was used to identify Siglec‐1 as a clinically relevant monocytes receptor. Our microarray data also showed that a considerable number of genes are downregulated in adult PBMCs following RSV exposure. However, none of these genes were significantly downregulated in vivo in whole blood from RSV‐infected infants (data not shown). Upregulation of Siglec‐1 on monocytes has been observed in patients with atherosclerosis, systemic sclerosis, rheumatoid arthritis, systemic lupus erythematous and human immunodeficiency virus infections 19, 20, 21, 22, 23. The common denominator in these studies appears to be type I interferons (IFNs). Our study showed an upregulation of multiple interferon regulated genes in whole blood from infants with RSV infection indicating that the induction of Siglec‐1 during RSV infection could be dependent on type I IFNs. Human experimental RSV infection models in adults have been performed 24 and could be used to confirm our results derived from RSV‐infected infants. Whole blood gene expression may not accurately reflect the inflammatory response at the site of infection but rather reflect inflammation in general. Therefore, we determined the In vitro expression of Siglec‐1 on monocytes after exposure of PBMCs to RSV directly. In addition, we measured the expression of Siglec‐1 after exposure of PBMCs to lipopolysaccharide (LPS), a bacterial ligand, and we observed no increase of Siglec‐1 expression after exposure to LPS (data not shown). These data are in line with previous literature 19 and suggest that the induction of Siglec‐1 on monocytes may be interferon‐ or viral‐specific rather than an induction due to inflammation in general. We decided to use adult PBMCs to evaluate the upregulation of Siglec‐1 in adults, because this model would allow us to compare mononuclear cells form newborns and adults In vitro and investigate Siglec‐1‐dependent immune signaling. The comparable RSV‐induced expression of Siglec‐1 on newborn and adult monocytes suggests a viral‐induced pathway that is already functional at birth. RSV‐induced expression of Siglec‐1 may be present on recruited monocytes and/or resident macrophages to regulate T cell functionality at the site of infection 9.

The functionality of Siglec‐1 during viral infection differs between viruses. Siglec‐1 has been implicated in cell entry of human immunodeficiency virus (HIV). Sialoglycans are present on the gp120 envelope of HIV and mediate cell entry via Siglec‐1. Little is known about cellular receptors for RSV and nucleolin has been proposed as an important receptor for RSV fusion protein to allow cell entry of epithelial cells 25. Our data indicate that Siglec‐1 is not required for RSV infection of monocytes.

In general, newborn CD4^+^ T cells produce low amounts of IFN‐γ compared to adults 26. We show that newborn CD4^+^ T cells are capable of producing comparable levels of IFN‐γ compared to adults but in a delayed fashion compared to adults. The formation of IFN‐γ‐producing cells upon RSV infection in newborn mice is also delayed and may underlie the susceptibility of young infants to severe RSV infections in vivo 3. We propose that RSV‐specific IFN‐γ‐producing memory T cells in adults rapidly produce IFN‐γ In vitro, whereas IFN‐γ production by newborn naïve T cells newborns is delayed. In our assay, depletion of CD4^+^ T cells reduced the production of IFN‐γ by CBMC and PBMC upon exposure to RSV, whereas depletion of CD8^+^ T cells did not. Because our model does not include epithelial cells and lung‐residential immune cells, CD8^+^ T cells may still contribute to the production of IFN‐γ during RSV infections in vivo. In addition, depletion of memory CD4^+^ T cells reduced the production of IFN‐γ in adults. The remaining induction of IFN‐γ may be due to incomplete depletion of memory CD4^+^ T cell as flow cytometry showed approximately 70–80% reduction in memory CD4^+^ T cells (data not shown). Alternatively, naïve T cells could also be responsible for the induction of IFN‐γ.

This study shows a distinct inhibitory effect of Siglec‐1 on the release of IFN‐γ by adult CD4^+^ T cells in contrast to newborns. The expression of Siglec‐1 is comparable between newborn and adult monocytes. The expression of Siglec‐1 can, therefore, not explain the differences in Siglec‐1‐dependent signaling between newborns and adult T cells. Siglec‐1 is the only siglec without a signaling motifs which suggests a mode of action via cell‐cell interaction 18. A major difference between newborn and adult T cells is the high percentage of memory CD4^+^ T cells in adult PBMCs. Our data indicate that the presence of memory CD4^+^ T cells is required for Siglec‐1 to have an inhibitory effect on RSV‐induced IFN‐γ release. CD43 is a highly sialylated receptor on T cells and has been identified as the major receptor for Siglec‐1 27. T cells from mice lacking CD43 are able to bind Siglec‐1, which suggests that in mice another receptor may also be involved 28. However, in humans only human CD43 has been proposed as the main receptor for Siglec‐1 27. We show that the expression of CD43 is lower on newborn naïve CD4^+^ T cells compared to adult naïve CD4^+^ T cells. In addition, we show a high expression of CD43 on adult differentiated T cells such as effector memory T cells and terminally differentiated T cells compared to adult naïve CD4^+^ T cells. Cross‐linking of CD43 is important for proper cytokine production 29 and Siglec‐1 may prevent this process. We hypothesize that newborn and adult naïve T cells produce IFN‐γ in response to RSV but their low expression of CD43 may prevent the inhibitory binding of Siglec‐1. Contrary, adult memory T cells have a high expression of CD43 that allows Siglec‐1 to exert an inhibitory effect on the release of IFN‐γ. The use of primary human cells and the absence of a commercially available CD43 blocking antibody warrants the use of other models, such as mouse models, for future research to demonstrate that Siglec‐1 directly inhibits the production of IFN‐γ during RSV infection through CD43. Clinically, these data suggest that the inhibitory effect of Siglec‐1 on T cells mainly affects memory T cells during reinfections. Mejias et al also observed an inhibitory effect of RSV on T cells during RSV infections in infants 30. Future research should determine at what time after birth this inhibitory effect takes place, because our data indicate that in certain circumstances Siglec‐1‐dependent inhibition of T cells is not present directly after birth.

CD43 can bind other receptors than Siglec‐1 such as MHC‐I 31. The low expression of CD43 on newborn CD4^+^ T cells and the inhibitory role of Siglec‐1 in adults could therefore have multiple implications on T cells during infectious diseases. The inhibitory effect of monocytes on T cells via Siglec‐1 may apply to other antigen‐presenting cells such as dendritic cells (DCs) and macrophages. During In vitro human rhino virus infections, DCs inhibit T cells via Siglec‐1 16, 21, 32. Future research to investigate the cell‐cell interaction between monocytes, DCs, macrophages and T cells would enhance our understanding of the regulatory role of antigen‐presenting cells during RSV infection. In vivo, Siglec‐1‐deficient mice have an enhanced T cell response to exosomal antigens indicating an inhibitory Siglec‐1‐dependent pathway in mice 33. Infection of Siglec‐1‐deficient mice with RSV may give more insight into the role of Siglec‐1 during RSV infection in vivo.

In conclusion, this study identified a regulatory pathway during RSV infection that involves Siglec‐1, monocytes and T cells. Our data show a delayed kinetic of RSV‐induced IFN‐γ by newborn CD4^+^ T cells compared to adult CD4^+^ T cells and an inhibitory role of Siglec‐1 on the production of IFN‐γ in adults. Insights into the distinct Siglec‐1‐dependent inhibition of IFN‐γ by RSV in adults compared to newborns and the low expression of CD43 in newborns will provide a better understanding of the antiviral immune response against RSV directly after birth compared to adults.

## Materials and methods

### Transcriptome analysis of whole blood from RSV‐infected infants and RSV‐stimulated PBMCs

Our group has published whole blood gene expression of RSV‐infected infants and healthy control infants. The methods are described by Jong et al. 34 and the microarray data was deposited in the ArrayExpress database under the access number E‐MTAB‐5195. Ìn addition, we previously published a microarray database regarding PBMCs from healthy adults that are untreated or stimulated with RSV. The methods have been described by our group previously 35 and this microarray data has been deposited in the NCBI Gene Expression Omnibus (GEO) database under GEO Series accession number (GSE59391) 35. We combined these two microarray databases for the current study. To first select clinically relevant genes, we used a cut‐off value of 4‐fold difference between RSV‐infected infants and healthy controls in the whole blood transcriptome analyses. A similar cut‐off value of 4‐fold difference was used for the microarray data published by Vissers et al. 35 to identify genes that are upregulated In vitro upon exposure of adult PBMCs to RSV. We then determined which genes were upregulated in both microarray analyses, being upregulated genes derived from whole blood from RSV‐infected infants and upregulated genes derived from In vitro RSV‐stimulated adult PBMCs, to identify clinically relevant genes that could be investigated in our model of PBMCs. Written informed consent was obtained from all donors or from all parents of patients. The study was approved by the Regional Committee on Research involving Human Subjects Arnhem‐Nijmegen.

### Virus culture

Green fluorescent protein (GFP)‐labeled RSV A2 (rgRSV30), kindly provided by Dr. M.E. Peeples, was cultured as previously described 35.

### Cell isolation and stimulation

Human newborn cord blood was collected from the umbilical cord after Cesarean section. Premature births and births to HIV‐positive mothers were excluded. Peripheral blood was collected from healthy adult volunteers. Experimental guidelines of the Regional Committee on Research involving Human Subjects Arnhem‐Nijmegen were observed. Newborn or adult blood was layered onto Ficoll‐Hypaque (GE Healthcare) to collect cord blood mononuclear cells (CBMCs) and peripheral blood mononuclear cells (PBMCs), respectively. When indicated, monocytes were purified via negative selection (Monocyte Isolation Kit II human; Miltenyi Biotec). CD4^+^ T cells, memory CD4^+^ T cells, CD8^+^ T cells or CD56^+^ natural killer (NK) cells were depleted from the mononuclear (MC) fraction using specific microbeads (Miltenyi Biotec), according to the manufacturer's instructions. MCs or monocytes were incubated at 5 × 10^5^ or 1 × 10^5^ cells/well respectively and incubated with RSV at an MOI 1 at 37⁰C and 5% C0_2_ for 48h or 5 days. When indicated, MCs were pre‐treated with anti‐Siglec‐1 monoclonal antibodies (HSn 7D2, ab18619; Abcam) for 1h at 37⁰C. Blocking experiments were performed with matching isotype controls (mouse IgG1 Isotype, eBioscience).

### Flow cytometry

To analyse expression of surface markers, MCs were incubated with fluorochrome‐labeled monoclonal antibodies for 30 min in the dark on ice and fluorescence intensity was measured using a LSR II flow cytometer (BD Biosciences). CD3 V500, CD3 Alexa Fluor 647, CD4 PerCP‐Cy5.5, CD8 APC‐H7, CD14 V500, CD14 Pe‐Cy7, CD27 BV510, CD43 FITC, CD45RA PeCy‐7, CD56 BV510 (BD Biosciences) and Siglec‐1 Alexa Fluor 647 (Sanbio) were used. Relative mean intensity fluorescence (MFI) of CD43 was calculated as follows: MFI stained/MFI unstained. GFP fluorescence was used to determine replication of (GFP)‐labeled RSV.

### Cytokine release

IFN‐γ concentrations were measured in culture supernatants by enzyme‐linked immunosorbent assay (ELISA) according to the manufacturer's instructions (Sanquin, Amsterdam, The Netherlands). The detection limit was 19 pg/mL. All conditions were measured in duplicate.

### Statistical analysis

Statistical analyses employed the Wilcoxon matched‐pairs signed rank test for paired analysis between two conditions and repeated measures ANOVA with Bonferroni's Multiple Comparison Test for paired analysis between more than two conditions. Comparison between newborns and adults employed Mann–Whitney U test. Comparison between more than two groups employed the Kruskal–Wallis test followed by Dunn's Multiple Comparison Test. Tests were considered significant if *p*<0.05. All statistical analyses were done with GraphPad Prism.

## Conflict of interest

The authors declare no financial or commercial conflict of interest.

**Abbreviations**
CBMC: cord blood mononuclear cells
DC: dendritic cells
ELISA: enzyme‐linked immunosorbent assay
GEO: gene expression omnibus
GFP: green fluorescence protein
HIV: human immunodeficiency virus
IFN: interferon
IFN‐γ: interferon gamma
LPS: lipopolysaccharide
MC: mononuclear cells
MFI: mean fluorescence intensity
MHC: major histocompatibility complex
NGI: netherlands genomics institute
NK: natural killer
PBMC: peripheral blood mononuclear cells
RSV: respiratory syncytial virus
Siglec‐1: sialic acid‐binding immunoglobulin‐type lectin 1

## Supporting information

Peer review correspondence
