# Supplementary material for: Siglec‐1 inhibits RSV‐induced interferon gamma production by adult T cells in contrast to newborn T cells
Source: Eur J Immunol. 2018 Jan 18;48(4):621–31. doi: 10.1002/eji.201747161 (PMC5947594; doi:10.1002/eji.201747161)
Supplement: Supplementary file 1 — Peer review correspondence [file EJI-48-621-s001.pdf]

European Journal of Immunology

Siglec-1 inhibits RSV-induced interferon gamma production by adult T Cells in contrast to newborn T cells

Jop Jans, Wendy W.J. Unger, Marloes Vissers, Inge M.L. Ahout, Inge Schreurs, Arthur Wickenhagen, Ronald de Groot, Marien I. de Jonge and Gerben Ferwerda.

Correspondence: Dr. Gerben Ferwerda, Laboratory of Pediatric Infectious Diseases, Radboud university medical center, 6500 HB Nijmegen, The Netherlands.

Review Timeline:	Submission date:	30-May-2017
	First Editorial decision:	27-Jun- 2017
	Revisions received:	29-Oct-2017
	Accepted:	08-Dec-2017

Handling Executive Committee member: Prof. Annette Oxenius

Please note that the correspondence below does not include the standard editorial instructions regarding preparation and submission of revised manuscripts, only the scientific revisions requested and addressed.

First Editorial Decision

27-Jun-2017

Dear Dr. Ferwerda,

Manuscript ID eji.201747161 entitled "SIGLEC-1 INHIBITS RSV-INDUCED INTERFERON GAMMA PRODUCTION BY ADULT T CELLS IN CONTRAST TO NEWBORNS" which you submitted to the European Journal of Immunology has been reviewed. The comments of the referees are included at the bottom of this letter.

A revised version of your manuscript that takes into account the comments of the referees will be reconsidered for publication. The Executive Editor strongly encourages you to onboard the comments of the referees by performing extensive re-analysis of the work, providing additional clarification on methodology as well as increasing the scope of discussion in addition to performing the experiments requested by Referee 1. Should you disagree with any of the referees' concerns, you should address this in your point-by-point response and provide solid scientific reasons for why you will not make the requested changes.

You should also pay close attention to the editorial comments included below. **In particular, please edit your figure legends to follow Journal standards as outlined in the editorial comments. Failure to do this will result in delays in the re-review process.**

Please note that submitting a revision of your manuscript does not guarantee eventual acceptance, and that your revision will be re-reviewed by the referees before a decision is rendered.

If the revision of the paper is expected to take more than three months, please inform the editorial office. Revisions taking longer than six months may be assessed by new referees to ensure the relevance and timeliness of the data.

Once again, thank you for submitting your manuscript to European Journal of Immunology and we look forward to receiving your revision.

Yours sincerely,
Eloho Etemire

On behalf of
Prof. Annette Oxenius

Dr. Eloho Etemire
Editorial Office
European Journal of Immunology
e-mail: ejied@wiley.com
www.eji-journal.eu

****

Reviewer: 1

Comments to the Author
The manuscript describes a number of interesting observations concerning interferon-gamma (IFN-γ) production in response to RSV. Using two microarray datasets, SIGLEC-1 was identified as upregulated in response to RSV exposure/infection. Both newborn and adult monocytes upregulate SIGLEC-1 following RSV exposure, thereby inhibiting IFN-γ production by CD4+ T-cells in adults; however, in newborns the ligand for SIGLEC-1 (CD43) is not expressed at a high level and this effect does not occur.

The authors are to be commended for their use of natural infection samples where possible.

Comments

1) Whole blood gene expression changes during RSV infection may poorly represent alterations in the infection site. This is particularly relevant given the low degree of re-circulation of monocytes and some T-cell populations following migration to an inflamed site. Are the observed upregulated genes specific to RSV, or markers of inflammation generally?

2) It is often inadvisable to directly compare infant whole blood to adult PBMCs. The relative abundance of polymorphonuclear (PMN) cells in infant blood (PMN are at much lower levels in adult PBMC) may dilute the expression signature. This may account for the small number of differentially expressed genes in infant whole blood samples. Conversely, paucity of PMNs in adult samples is very likely responsible for the lack of MMP8 (neutrophil associated) upregulation in PBMC samples, which has been noted elsewhere (including by the present authors).

3) A considerable number of genes are downregulated in adult PBMCs following RSV exposure yet these are not discussed. Do these include any suppressors of T cell activity?

4) Adult PBMCs respond to RSV by 48h. Is the response greater at 5d, or has it ended?

5) How many receptors on T-cells are 'highly sialylated'? (Line 134). CD43 is not the only candidate for SIGLEC-1 binding; alternative should be discussed.

6) The authors demonstrate that CBMCs have delayed IFN-γ production, compared to adults, and that this is generated by CD4 cells in both adults and infants. SIGLEC-1 is induced during RSV infection in adults and infants. SIGLEC-1 is then demonstrated to suppress IFN-γ in adult PBMCs (not infant CBMBCs) possibly due to higher CD43 expression. Presumably the difference in kinetics between infant and adult CD4 cell IFN-γ generation is due to central memory cells (as mentioned in discussion), this should be demonstrated directly i.e. do naïve CD4 cells from infants and adults behave identically in terms of kinetic expression of IFN-γ, but adults are suppressed in the scale of this response? The results are presumably strongly affected by memory cells.

7) IFN-γ production is reported to be solely due to CD4 T-cells in culture experiments. This may not be the case in natural infection, where CD8 T-cells and natural killer cells are considered major sources of IFN-γ. This discrepancy may be due to the experimental system being used; RSV antigens may be being presented by blood antigen presenting cells in a MHC-II dependent manner, resulting in a CD4 biased response. By contrast in natural infection, infection of RSV target cells, such as epithelial cells, results in a broader immunological response involving CD8 T-cells and NK cells. This limits the biological relevance of the observations, and might be mentioned in the discussion.

8) Is SIGLEC-1 upregulated by the virus to suppress the immune response? Or is this an effect of signals from infected cells? Many genes from infant samples, including SIGLEC-1, are ISGs, suggesting the latter. This is mentioned in the discussion but not explored. Why would the immune response suppress type-II IFN production (through type-I IFN)? Is induction of type-I IFNs equivalent between CBMCs and PBMCs? This would strengthen the argument.

9) Too much emphasis is given to the higher expression of CD43 in adult MC samples. Without evidence directly

demonstrating that CD43 is the crucial receptor for SIGLEC-1 mediated inhibition of IFN-γ production, the mechanism is incomplete. The author's use of human samples is commendable, but in this instance a CD43 knockdown, or antibody mediated inhibition experiment is warranted.

10) The conclusion (line 201-202) that adult memory cells are suppressed by SIGLEC-1 due to high CD43 expression (not seen in naïve infant T cells) is questionable. Central memory CD4 T cells from adults are not directly comparable to naïve infant T cells from cord blood. The authors should ideally compare naïve T cells from both infants and adults to show that an age-dependent effect of SIGLEC-1 on IFN-γ production is mediated by CD43.

Suggested typographical, statistical and presentation improvements:

- Sentence on lines 56-58 should be altered to "Inhibition or inadequate activation of T cells, resulting in lower IFN-γ secretion, may explain the high rate of reinfections" or similar.
- Adjust the Y-axis size on figures 3a and 3b.
- The sentence ending in line 199 does not make sense.
- The statistical tests employed for figure 1d are not clear. These should be corrected for multiple testing but the legend states 'followed by Mann-Whitney U test'. This seems inappropriate.
- An addition to figure 5 with back-gating of CD43 expression to demonstrate the non-CD4+ cells that express CD43 is warranted.

In conclusion, the manuscript presents some interesting data; however, much of this is potentially confounded or incomplete.

Examples of this are detailed in the Comments section, the major points of which are:

1) Comparing gene expression data from infant whole blood and adult in vitro stimulated PBMCs is fraught with problems.

2) The kinetics of IFN-γ production are not fully explored.

3) Naïve T-cells should be analysed specifically, from both adults and infants.

4) The generation of IFN-γ by CD4 T-cells may be an artefact of tissue culture and should be interpreted with great caution in the light of other publications.

5) CD43 is not conclusively demonstrated to be the SIGLEC-1 receptor of relevance to IFN-γ production.

Reviewer: 2

Comments to the Author

In their manuscript "Siglec-1 inhibits RSV-induced interferon gamma production by adult T cells in contrast to newborns", Jans et. al demonstrate that Siglec-1 is upregulated on MC and monocytes following RSV infection. Coculture of infected monocytes with adult PBMC blocked release of IFNg, which could be reversed by blocking Siglec-1 with a specific antibody, while the same was not true for cord blood mononuclear cells. The authors

propose that this age-dependence is due to a higher frequency of antigen-experienced T cells, and higher CD43 expression on adult PBMC.

Major issues:

1. The transcriptome analysis section is not at all clear. The number of subjects analyzed and methods of analysis are not clear. The merging of the two major data sets and comparisons are not laid out in an easy to understand way. No healthy infants are mentioned in the methods, and it is not clear if the “recovery” group for Figure 1D is composed of the same individuals as the “acute” group.

2. Methods are not provided for the RSV titer data in Figure 1C. Assuming that CT value means the cycle threshold, it would make sense to try the correlation analysis after removal of the two samples that appear to have a Ct over 35.

Minor issues:

1. For the title, I would recommend changing “in contrast to newborns” to “in contrast to those of newborns”, or “in contrast to newborn T cells”.

2. The methods are often not very clear about incubation times (for example how long were monocytes infected prior to blocking siglec and coincubation with T cells?) among other things (RSV titer measurements, etc).

3. The graph on the bottom left of Figure 2 appears to be a repeat of Figure 2B.

First Revision – authors' response

29-Oct-2017

Reviewer comments:

Reviewer #1:

The manuscript describes a number of interesting observations concerning interferon-gamma (IFN-γ) production in response to RSV. Using two microarray datasets, SIGLEC-1 was identified as upregulated in response to RSV exposure/infection. Both newborn and adult monocytes upregulate SIGLEC-1 following RSV exposure, thereby inhibiting IFN-γ production by CD4+ T-cells in adults; however, in newborns the ligand for SIGLEC-1 (CD43) is not expressed at a high level and this effect does not occur. The authors are to be commended for their use of natural infection samples where possible.

First of all, we would like to thank the reviewer for the constructive comments and the appreciation of our manuscript and our use of natural infection samples where possible.

European Journal of Immunology

Comments

1. Whole blood gene expression changes during RSV infection may poorly represent alterations in the infection site. This is particularly relevant given the low degree of re-circulation of monocytes and some T-cell populations following migration to an inflamed site. Are the observed upregulated genes specific to RSV, or markers of inflammation generally?

We agree with the reviewer that blood gene expression during RSV infection may not accurately represent alteration in the infection site. In our manuscript, we used the whole blood gene expression to select for genes that are upregulated during in vivo RSV infections. Indeed, we cannot exclude the effects of inflammation in general on in vivo gene expression. However, we performed additional experiments to determine whether Siglec-1 is induced in vitro by bacterial ligands as well. LPS, a bacterial ligand that induces TLR4 signaling, did not induce Siglec-1 on monocytes (data not shown). These results are in line with previous literature that show IFN-dependent induction of Siglec-1 rather than induction by inflammation in general (York et al; Arthritis & Rheumatism 2007). We have added a section in our discussion section to properly reflect on the applicability of whole blood gene expression during RSV infections and these additional experiments and results (line 250-259).

2. It is often inadvisable to directly compare infant whole blood to adult PBMCs. The relative abundance of polymorphonuclear (PMN) cells in infant blood (PMN are at much lower levels in adult PBMC) may dilute the expression signature. This may account for the small number of differentially expressed genes in infant whole blood samples. Conversely, paucity of PMNs in adult samples is very likely responsible for the lack of MMP8 (neutrophil associated) upregulation in PBMC samples, which has been noted elsewhere (including by the present authors).

The direct comparison of whole blood with adult PBMC will indeed be affected by the presence of PMN in whole blood. Therefore, we only used this approach to identify gene candidates that would be upregulated during in vivo infection. We subsequently confirmed these results in our in vitro model of CBMC and PBMC to create a more accurate comparison between the two age groups.The majority of the manuscript investigated the mechanisms behind the induction of IFN-γ and the role of Siglec-1. In all these experiments, we made a comparison between CBMCs and PBMCs and excluded confounding factors introduced by other immune cells that are present in whole blood.

3. A considerable number of genes are downregulated in adult PBMCs following RSV exposure yet these are not discussed. Do these include any suppressors of T cell activity?

We agree with the reviewer that a considerable number of genes are downregulated in adult PBMCs following RSV exposure. Similar to our analysis with upregulated genes, we compared these downregulated genes with the microarray derived from RSV-infected infants to determine whether these genes are also downregulated in vivo. However, none of the genes that are downregulated in PBMCs are downregulated in vivo in whole blood from RSV-infected infants (data not shown). Therefore, we have not included these data in our manuscript. We have included a section our manuscript to inform the reader about this analysis (line 239-242).

4. Adult PBMCs respond to RSV by 48h. Is the response greater at 5d, or has it ended?

We performed additional experiments and exposed adult PBMCs to RSV for 5 days. In adults, IFN-γ is still present in the supernatant after 5 days exposure to RSV. The amount of IFN-γ after 5 days is comparable to the amount of IFN-γ produced by adult PBMCs after exposure to RSV for 48h (Figure 3B). We have included these data in the manuscript in the result section (line 169-170).

5. How many receptors on T-cells are ‘highly sialylated’? (Line 134). CD43 is not the only candidate for SIGLEC-1 binding; alternative should be discussed.

Siglec-1 is known as sialic-acid binding Ig like lectin , because of its binding to sialic acid-containing receptors. It is important to note that Siglec-1 binds different receptors dependent on cell type and, in addition, does not bind all sialylated receptors. Van de Berg et al investigated the binding of Siglec-1 on T cells [Van den Berg 2001, J Immunol]. Via Sialoadhesin-Fc fusion proteins and immunoprecipitations, only two potential receptors on T cells

European Journal of Immunology

were found: CD43 and CD162. In subsequent experiments, the authors show that overexpression of CD43 increased Siglec-1 binding, whereas overexpression of CD162 did not. The authors conclude that CD43 is a major counterreceptor for Siglec-1 on T cells. Kidder et al showed that Siglec-1 is able to bind T cells that lack CD43 and CD162 expression in mice, which suggest that other sialylated receptors are also involved [Kidder 2013 Immunol]. However, no specific receptor other than CD43 has been identified up to now. Although literature is scarce, we believe that this paper by Van den Berg et al provided compelling evidence that CD43 is a target for Siglec-1 on T cells. We have included a section in the discussion regarding this topic (line 296-299 and line 300-303).

6. The authors demonstrate that CBMCs have delayed IFN-γ production, compared to adults, and that this is generated by CD4 cells in both adults and infants. SIGLEC-1 is induced during RSV infection in adults and infants. SIGLEC-1 is then demonstrated to suppress IFN-γ in adult PBMCs (not infant CBMBCs) possibly due to higher CD43 expression. Presumably the difference in kinetics between infant and adult CD4 cell IFN-γ generation is due to central memory cells (as mentioned in discussion), this should be demonstrated directly i.e. do naïve CD4 cells from infants and adults behave identically in terms of kinetic expression of IFN-γ, but adults are suppressed in the scale of this response? The results are presumably strongly affected by memory cells.

We agree with the reviewer that the delayed IFN-y production in newborns is most likely due to the absence of CD4$^+$ memory T cells. We performed additional experiments in which we depleted adult PBMCs from memory CD4$^+$ T cells. As expected, depletion of memory CD4$^+$ T cells reduced RSV-induced IFN-γ production (line 191-192; Figure 4E). The remaining induction of IFN-γ may be due to incomplete depletion of memory CD4$^+$ T cell as flow cytometry showed approximately 70-80% reduction in memory CD4$^+$ T cells (data not shown). Alternatively, naïve T cells could also be responsible for the induction of IFN- γ. However, the scope of our manuscript was investigate the effect of Siglec-1 on the production of IFN-γ and not to determine the exact subset of CD4$^+$ T cell involved in the production of IFN-γ. Therefore, we used microbeads to gain more insight into the contribution of memory CD4$^+$T cells. Future research should investigate in depth which CD4$^+$ T cell is the main producer of IFN-γ. We have include a section in the discussion to contemplate on these results (line 285-290).

7. IFN-γ production is reported to be solely due to CD4 T-cells in culture experiments. This may not be the case in natural infection, where CD8 T-cells and natural killer cells are considered major sources of IFN-γ. This discrepancy may be due to the experimental system being used; RSV antigens may be being presented by blood antigen presenting cells in a MHC-II dependent manner, resulting in a CD4 biased response. By contrast in natural infection, infection of RSV target cells, such as epithelial cells, results in a broader immunological response involving CD8 T-cells and NK cells. This limits the biological relevance of the observations, and might be mentioned in the discussion.

The presence of CD8$^+$ T cells is indeed important during in vivo RSV infections. In the discussion, we have added an additional section to discuss our results regarding CD8$^+$ T cells and the possibility of skewing due to the experimental in vitro system (line 280-285).

8. Is SIGLEC-1 upregulated by the virus to suppress the immune response? Or is this an effect of signals from infected cells? Many genes from infant samples, including SIGLEC-1, are ISGs, suggesting the latter. This is mentioned in the discussion but not explored. Why would the immune response suppress type-II IFN production (through type-I IFN)? Is induction of type-I IFNs equivalent between CBMCs and PBMCs? This would strengthen the argument.

Our group has previously published data on the induction of type I IFNs by CBMCs and PBMCs [Jans et al, 2016, J Allergy and Clin Immunol]. We have shown that the induction of IFN-α by RSV is reduced in CBMCs compared to PBMCs, whereas the production of IFN-β after exposure to RSV is similar between newborns and adults. We have performed additional experiments in the current study and show that both IFN-α and IFN-β induce Siglec-1 on newborn and adult monocytes (data not shown) which is in line with previous literature (York et al; Arthritis & Rheumatism 2007). Therefore, we hypothesize that RSV induces type I INFs that lead to the expression of Siglec-1 to suppress IFN-y production by adult T cells. Clinically, the suppression of IFN-y production by type I IFNs may be beneficial to control the inflammatory response during RSV infections. We decided not to include the induction of Siglec-1 by type I IFNs in our manuscript, because it has already been published by York et al (line 255-259)

European Journal of Immunology

9) Too much emphasis is given to the higher expression of CD43 in adult MC samples. Without evidence directly demonstrating that CD43 is the crucial receptor for SIGLEC-1 mediated inhibition of IFN-γ production, the mechanism is incomplete. The author's use of human samples is commendable, but in this instance a CD43 knockdown, or antibody mediated inhibition experiment is warranted.

We agree with the reviewer that our use of human samples is one of the important strengths of our manuscript. Unfortunately, the use of human samples also makes it difficult to use CD43 knockdown cells. In addition, a CD43 blocking antibody that can be used in antibody-mediated inhibition experiments is currently not available. Therefore, we cannot directly link our phenotype with CD43 expression. Nevertheless, we believe our results should lead to future experiments that can connect the suppressive effect of RSV-induced Siglec-1 with CD43 expression. We have included an additional paragraph in our discussion section to warrant such research that most likely could occur in mice (line 321-324). In addition, we reduced the emphasis of the high expression of CD43 as mechanism of the Siglec-1 mediated inhibition of IFN-γ.

10) The conclusion (line 201-202) that adult memory cells are suppressed by SIGLEC-1 due to high CD43 expression (not seen in naïve infant T cells) is questionable. Central memory CD4 T cells from adults are not directly comparable to naïve infant T cells from cord blood. The authors should ideally compare naïve T cells from both infants and adults to show that an age-dependent effect of SIGLEC-1 on IFN-γ production is mediated by CD43.

We agree with the reviewer that ideally adult naïve T cells should be compared to newborn T cells to investigate the effect of Siglec-1 on the production of IFN-γ. Therefore, we performed additional experiments in which we depleted adult PBMCs from memory CD4$^+$ T cells. We subsequently stimulated these PBMCs (depleted of memory CD4$^+$ T cells) with RSV in the presence of absence of Siglec-1 blocking antibodies. Our results show that Siglec-1 no longer has an inhibitory effect on the production of IFN-γ by PBMCs after exposure to RSV when memory CD4$^+$ T cells are depleted from the experiment (line 189-194; Figure 4F). These data confirm our previously mentioned hypothesis that Siglec-1 only inhibits (adult) memory CD4$^+$ T cells and their production of IFN-γ, whereas the production of IFN-γ by naïve adult and newborn CD4$^+$ T cells are not inhibited by Siglec-1.We have included an additional figure containing these results (Figure 4F). In addition, we adjusted the methods, the results and the discussion section, respectively line 397, line 189-194 and line 296-299.

Suggested typographical, statistical and presentation improvements:

- Sentence on lines 56-58 should be altered to "Inhibition or inadequate activation of T cells, resulting in lower IFN-γ secretion, may explain the high rate of reinfections" or similar.
- Adjust the Y-axis size on figures 3a and 3b.
- The sentence ending in line 199 does not make sense.
- The statistical tests employed for figure 1d are not clear. These should be corrected for multiple testing but the legend states 'followed by Mann-Whitney U test'. This seems inappropriate.
- An addition to figure 5 with back-gating of CD43 expression to demonstrate the non-CD4+ cells that express CD43 is warranted.

We thank the reviewer for the suggested typographical, statistical and presentation improvement and we have adjusted our manuscript accordingly. We agree that figure 1D should be corrected for multiple testing. We changed the statistical analysis and performed a Kruskall Wallis test with Dunn's Multiple Comparison test as correction for multiple testing. We have changed the description of the statistical analysis accordingly. We did not perform an extensive staining for other cells such as monocytes, NK cells or CD8$^+$ T cells to determine the expression of CD43 on other immune cells. Because the expression of CD43 varies between immune cells such as CD8$^+$ T cells or monocytes, a broad range of mean fluorescence intensity would be seen when back-gating for non CD4$^+$ cells. We, therefore, believe that back-gating of CD43 expression would not provide valuable data for the scope of this manuscript.

Reviewer #2

Comments to the Author
In their manuscript "Siglec-1 inhibits RSV-induced interferon gamma production by adult T cells in contrast to newborns", Jans et. al demonstrate that Siglec-1 is upregulated on MC and monocytes following RSV infection.

European Journal of Immunology

Coculture of infected monocytes with adult PBMC blocked release of IFNg, which could be reversed by blocking Siglec-1 with a specific antibody, while the same was not true for cord blood mononuclear cells. The authors propose that this age-dependence is due to a higher frequency of antigen-experienced T cells, and higher CD43 expression on adult PBMC.

First of all, we would like to thank the reviewer for the constructive comments and critical appraisal of our manuscript.

Major issues:

1. The transcriptome analysis section is not at all clear. The number of subjects analyzed and methods of analysis are not clear. The merging of the two major data sets and comparisons are not laid out in an easy to understand way. No healthy infants are mentioned in the methods, and it is not clear if the "recovery" group for Figure 1D is composed of the same individuals as the "acute" group.

We agree with the reviewer that the transcriptome analysis is not fully explained. We have extended the specific method section to increase the clarity of our experimental design. The number of subject were already mentioned in Table 1. We included the number of subjects in the figure legends (line 553-554 and line 563). We referred to previous publications from our group in which a detailed description is provided regarding the analysis of the whole blood gene expression of RSV-infected infants (Jong et al 2016, Scientific Reports) and the analysis of PBMCs exposure to RSV (Vissers et al 2015, Cytokine). If warranted, we can copy that description into our manuscript. From the recovery samples, gene expression from 8 individuals could be directly compared with the gene expression during the acute phase of infection. In all cases, the expression of Siglec-1 was higher compared to the expression of Siglec-1 during the recovery phase 4-6 weeks after infections (data not shown). We have added a paragraph in the results section to describe these results (line 100-102). We adjusted the part regarding the merging of the two major data sets and created a more comprehensive description (line 84-93)

2. Methods are not provided for the RSV titer data in Figure 1C. Assuming that CT value means the cycle threshold, it would make sense to try the correlation analysis after removal of the two samples that appear to have a Ct over 35.

The cut off for Ct value is debatable. Indeed, the RSV titer data is depicted as Ct values and we did not use a cut off value. We agree that the removal of samples with a Ct value over 35 reduced the chance of including false positive Ct values. Therefore, we have removed the sample with a Ct value over 35 and re-analyzed the data. We still observed a significant negative correlation between Ct value and Siglec-1 expression as depicted in Figure 1C (Figure 1C)

Minor issues:

1. For the title, I would recommend changing "in contrast to newborns" to "in contrast to those of newborns", or "in contrast to newborn T cells".

We have changed the title accordingly.

2. The methods are often not very clear about incubation times (for example how long were monocytes infected prior to blocking siglec and coincubation with T cells?) among other things (RSV titer measurements, etc).

We have provided clarification in our method section with regard to incubation times, such as blocking Siglec-1 and exposure to RSV, and RSV titer measurements (line 401 and line 402).

3. The graph on the bottom left of Figure 2 appears to be a repeat of Figure 2B.

We agree with the reviewer that due to editing errors on our side a repeat of Figure 2B was accidently included. We have now included the correct figure.

European Journal of Immunology

Second Editorial Decision
30-Nov-2017

Dear Dr. Ferwerda,

It is a pleasure to provisionally accept your manuscript entitled "SIGLEC-1 INHIBITS RSV-INDUCED INTERFERON GAMMA PRODUCTION BY ADULT T CELLS IN CONTRAST TO NEWBORN T CELLS" for publication in the European Journal of Immunology. For final acceptance, please follow the instructions below and return the requested items as soon as possible as we cannot process your manuscript further until all items listed below are dealt with.

Please note that EJI articles are now published online a few days after final acceptance (see Accepted Articles: http://onlinelibrary.wiley.com/journal/10.1002/(ISSN)1521-4141/accepted). The files used for the Accepted Articles are the final files and information supplied by you in Manuscript Central. You should therefore check that all the information (including author names) is correct as changes will NOT be permitted until the proofs stage.

We look forward to hearing from you and thank you for submitting your manuscript to the European Journal of Immunology.

Yours sincerely,
Marta Vuerich

on behalf of
Prof. Annette Oxenius

Dr. Marta Vuerich
Editorial Office
European Journal of Immunology
e-mail: ejied@wiley.com
www.eji-journal.eu
